# Resting-state functional connectivity of the sensorimotor network in medication-naïve Chinese children with ADHD: cross-sectional associations with hyperactivity/impulsivity and executive function

**DOI:** 10.3389/fpsyt.2026.1843959

**Published:** 2026-07-27

**Authors:** Han-Lin Li, Meng-Jie Zhang, Teng Zhu, Jia-Yun Yu, Jia-Qi Zhou, Chuang Yang

**Affiliations:** 1 Department of Psychiatry, Beilun District People's Hospital, Ningbo, Zhejiang, China; 2 Department of Psychiatry, Wenzhou Seventh People’s Hospital, Wenzhou, China; 3 Department of Psychiatry, The First Affiliated Hospital of Wenzhou Medical University, Wenzhou, China

**Keywords:** ADHD, executive function, functional connectivity, medication-naïve children, sensorimotor network

## Abstract

**Background:**

Attention-deficit/hyperactivity disorder (ADHD) is a prevalent neurodevelopmental disorder, often—but not always—characterized by inattention, hyperactivity/impulsivity, and deficits in executive functions (EFs); some individuals may also exhibit sensorimotor dysfunction. While SMN dysfunction has been implicated in ADHD, the patterns of SMN functional connectivity (FC) and their relationships with clinical symptoms and EFs remain inconsistent, partly due to methodological heterogeneity.

**Methods:**

The study included 62 medication-naïve patients with ADHD (aged 6–15 years) and 46 healthy controls matched for age, sex, and IQ. Clinical symptoms and EFs were assessed using CPRS, IVA-CPT, and Stroop tests. Resting-state fMRI data were acquired, and ROIs within SMN, SN, DMN, and FPN were selected from the Dosenbach atlas. FC was analyzed using Network-Based Statistics (NBS). Partial correlations explored FC–behavior relationships in the ADHD group, controlling for age, sex, and IQ.

**Results:**

The ADHD group showed significantly higher CPRS hyperactivity/impulsivity scores and poorer IVA-CPT and Stroop performance. Compared to controls, ADHD showed increased FC within SMN (right posterior insula to left precentral gyrus/parietal lobe), between SMN–DMN (precentral gyrus, dorsal frontal cortex, temporal lobe, angular gyrus, posterior cingulate cortex, and precuneus), and SMN–SN (middle insula, superior parietal lobule, superior temporal gyrus, basal ganglia, and fusiform gyrus). Enhanced intra-SMN FC was negatively correlated with impulsivity/hyperactivity and hyperactivity index scores. Enhanced SMN–DMN FC was negatively correlated with hyperactivity symptoms and positively correlated with control quotients in IVA-CPT. Enhanced SMN–SN FC was negatively correlated with Stroop correct responses and positively correlated with omission errors.

**Conclusions:**

In this medication-naïve pediatric sample, distinct SMN connectivity patterns differentially related to ADHD symptoms and executive function. However, given the cross-sectional design and modest sample size, these associations cannot establish causality; they require replication in longitudinal studies and larger cohorts to clarify whether they reflect developmental variation, neurobiological subtypes, or epiphenomena.

## Introduction

Attention-deficit/hyperactivity disorder (ADHD) is a prevalent neurodevelopmental disorder affecting approximately 5% of children globally and 6.26% in China (1, 2), often—but not always—characterized by inattention, hyperactivity/impulsivity, and deficits in executive functions (EFs) such as response inhibition and working memory (3, 4). Beyond these core features, mounting evidence indicates that some individuals with ADHD may also exhibit sensorimotor dysfunction, including deficits in sensory input, motor output, and sensorimotor integration (5, 6). For example, South African primary school children with ADHD have shown impaired fine motor skills (5), and Belgian children with ADHD and autism spectrum disorder displayed motor abnormalities associated with regional gray matter volumes (6). These sensorimotor deficits are not merely peripheral; they interact with higher-order cognitive processes, suggesting that the sensorimotor network (SMN) may play a critical role in the pathophysiology of ADHD.

Neuroimaging studies have consistently identified anatomical and functional alterations within sensorimotor regions in ADHD (7–9). For instance, U.S. children with ADHD showed atypical motor and sensory cortex activation during finger tapping (7), Canadian adolescents and adults with ADHD exhibited altered cortical morphology in sensorimotor processing regions (8), and Chinese youth with ADHD demonstrated distinct cortical thickness alterations (9). Early work in drug-naïve South Korean boys (aged 7–12 years) documented altered regional cerebral blood flow in bilateral sensorimotor cortices (10), whereas Chinese boys of the same age range showed increased amplitude of low-frequency fluctuations in the left sensorimotor cortex (11). More recent investigations using magnetoencephalography in Canadian adults with ADHD revealed atypical oscillatory activity (12), and resting-state fMRI studies in Chinese ADHD patients identified abnormal dynamic brain entropy within the SMN (13). Structurally, the SMN encompasses core sensorimotor cortices (precentral and postcentral gyri, supplementary motor area) as well as associated regions in the frontal, parietal, and insular cortices that collectively mediate sensorimotor integration and motor control (14, 15). Genetic and neurophysiological evidence also links SMN dysfunction to the severity of hyperactive/impulsive symptoms and inhibitory control deficits (16, 17). Specifically, Chinese early adolescents with ADHD showed multimodal SMN alterations predictive of diagnosis (16), and Chinese children carrying the DRD4 2-repeat allele exhibited prefrontal cortex network abnormalities related to SMN function (17).

Despite these advances, the functional connectivity (FC) patterns of the SMN in ADHD remain incompletely characterized and inconsistently reported. Some studies observe increased intra-SMN or SMN–default mode network (DMN) connectivity associated with milder symptoms or better EF (18, 19). For example, Chinese children with ADHD (N > 60) demonstrated that increased dynamic FC between DMN and SMN was associated with reduced symptoms (18), and Chinese children with ADHD showed altered neurovascular coupling in SMN regions (19). Conversely, other studies report reduced connectivity between sensorimotor and striatal regions correlated with motor overflow and poor integration (20). Notably, Canadian children with ADHD and developmental coordination disorder (half of whom were on psychostimulants) exhibited reduced FC between M1 and somatosensory/striatal regions (20). These discrepancies likely reflect methodological diversity—including seed-based versus independent component analysis (ICA) approaches, varying age ranges (children vs. adults), medication status, and motion-correction strategies—as well as the inherent developmental heterogeneity of brain networks (21). Notably, a recent systematic review and meta-analysis of rs-fMRI studies in ADHD highlighted substantial methodological variability across sites and emphasized the need for carefully controlled, medication-naïve pediatric samples to improve reproducibility (21). Whether increased or decreased SMN connectivity represents a pathological signature, a compensatory adaptation, or a neurobiological subtype remains unresolved, particularly in drug-naïve children. As summarized in **Table 1**, neuroimaging findings in ADHD indicate that SMN functional activity and connectivity vary by developmental stage, clinical status, and methodological approach.

**Table 1:** Related research on sensorimotor network.

Study	Population/sample	Age and clinical status	Design/method	Key findings related to SMN
Lee et al. (2005) (10)	Drug-naïve boys with ADHD (South Korea)	7–12 years; medication-naïve	Resting-state rCBF (SPECT)	Altered rCBF in bilateral sensorimotor areas
Zang et al. (2007) (11)	Boys with ADHD (China)	7–12 years; medication-naïve	rs-fMRI (ALFF)	Increased ALFF in left sensorimotor cortex
Tian et al. (2008) (22)	Children with ADHD (China)	7–14 years; primarily boys	rs-fMRI (RSAI)	Higher RSAI in left sensory cortex at rest
Dockstader et al. (2009) (12)	Adults with ADHD (Canada)	Adults; clinical diagnosis	MEG during task	Reduced somatosensory regulation and responsiveness
McLeod et al. (2014) (20)	Children with ADHD/DCD (Canada)	Children; 50% on psychostimulants	rs-fMRI (seed-based)	Reduced FC between M1 and somatosensory/striatal regions
Lin et al. (2018) (23)	Drug-naïve adults with ADHD	>80 adults; medication-naïve	rs-fMRI (NBS)	Enhanced SMN–SN FC negatively correlated with cognitive EF
Vatansever et al. (2019) (24)	Healthy young adults (UK)	~200 young adults; non-clinical	rs-fMRI	Reduced sensorimotor–DMN FC correlated with subclinical ADHD symptoms
Sörös et al. (2019) (25)	Adults with ADHD (Germany)	21–61 years; predominantly female	rs-fMRI (dimensional)	Increased intra-SMN FC correlated with hyperactivity severity
Chen et al. (2021) (26)	Children with ADHD vs. HC	8–12 years	rs-fMRI (ICA)	Increased inter-hemispheric SSN FC; linked to motor overflow
Sun et al. (2021) (27)	Boys with ADHD (China)	School-age boys	rs-fMRI (dFC)	SMN–DMN–FPN dFC abnormalities linked to EF deficits
Luo et al. (2023) (18)	Children with ADHD (China)	>60 children	rs-fMRI (dFC)	Increased DMN–SMN dFC associated with milder symptoms
Schwarzlose et al. (2023) (28)	Children with sensory over-responsivity (SOR)	Large multi-site pediatric sample	rs-fMRI	Increased SMN–SN FC; SOR severity correlated with ADHD symptoms

The present study addresses these gaps by examining resting-state FC within the SMN and between the SMN and three large-scale networks implicated in ADHD pathophysiology—the DMN, salience network (SN), and frontoparietal network (FPN)—in medication-naïve Chinese children with ADHD. We hypothesized that, compared to healthy controls (HCs), children with ADHD would exhibit altered FC within the SMN and between the SMN and EF-related networks (DMN, SN), and that these alterations would be associated with the severity of hyperactivity/impulsivity symptoms and response inhibition deficits.

## Materials and methods

### Participants and clinical assessment

This study was approved by the Ethics Committee of the First Affiliated Hospital of Wenzhou Medical University. Written informed consent was obtained from all guardians. We recruited 122 right-handed children and adolescents aged 6–15 years. Seventy-three medication-naïve patients meeting DSM-5 criteria for ADHD were diagnosed by two attending psychiatrists via the Schedule for Affective Disorders and Schizophrenia for School-Age Children-Present and Lifetime Version (K-SADS-PL). Forty-nine HCs matched for age, sex, and IQ were recruited from local schools. Exclusion criteria for both groups were: (i) comorbid psychiatric disorders (except learning disabilities); (ii) history of substance abuse, major head trauma, or neurological disorder; (iii) full-scale IQ ≤ 90; (iv) prior exposure to psychotropic medication; and (v) MRI contraindications or structural brain abnormalities.

Clinical and cognitive assessments included the Chinese version of the Conners’ Parent Rating Scale–Revised (CPRS-R) (29), the Integrated Visual and Auditory Continuous Performance Test (IVA-CPT) (30), the Stroop Color-Word Test (31), the Wechsler Intelligence Scale for Children–Revised (WISC-R) (32), and the Edinburgh Handedness Inventory (EHI) (33).

### MRI data acquisition

Resting-state fMRI data were acquired on a 3.0T GE scanner using a gradient-echo EPI sequence: TR = 2000 ms, TE = 30 ms, flip angle = 90°, 31 axial slices (4 mm thickness, 0.2 mm gap), matrix = 64 × 64, FOV = 192 × 192 mm². Each run comprised 240 volumes. Participants were instructed to lie still with eyes closed, remain awake, and avoid systematic thinking. Earplugs and foam padding were used to minimize noise and head motion.

### Data preprocessing and ROI definition

Imaging data were preprocessed using DPARSF v5.4 (34) (http://www.restfmri.net) in MATLAB. The pipeline included: (i) DICOM-to-NIFTI conversion; (ii) discarding the first 10 volumes for magnetization equilibrium; (iii) slice-timing correction; (iv) head-motion correction (translation > 3 mm or rotation > 3° excluded), with Friston-24 parameters and mean framewise displacement (mean FD) calculated (threshold < 0.3 mm); (v) co-registration to structural images; (vi) spatial normalization to MNI space via DARTEL (resampled to 3 × 3 × 3 mm^3^); (vii) spatial smoothing (6 mm FWHM Gaussian kernel); (viii) linear detrending; (ix) band-pass filtering (0.01–0.1 Hz); and (x) nuisance regression of white matter, cerebrospinal fluid, and Friston-24 motion parameters.

Regions of interest (ROIs) were selected from the Dosenbach atlas (15). To focus on networks central to sensorimotor and executive function, we included 120 of 160 ROIs from the SMN (n = 33), DMN (n = 34), FPN (n = 21), and SN (n = 32), excluding the cerebellar and occipital/visual networks due to limited anatomical coverage and reduced signal-to-noise ratio in these regions, as well as their limited relevance to the executive function and attention networks central to ADHD pathophysiology (35). For each ROI, a 5-mm radius sphere was constructed to balance spatial specificity and signal-to-noise ratio, consistent with prior ADHD connectivity studies (36).

### Functional connectivity and statistical analyses

Mean time series were extracted from each ROI, and Fisher z-transformed Pearson correlation coefficients were computed to generate a 120 × 120 ROI-to-ROI FC matrix for each participant. Group-level differences in network connectivity were assessed using Network-Based Statistics (NBS) v1.2 (37) (https://www.nitrc.org/projects/nbs/). A general linear model (GLM) was fitted for each edge with group (ADHD vs. HC) as the predictor of interest, controlling for sex, age, and full-scale IQ as nuisance covariates. Two-sided t-contrasts were thresholded at t > 3.0 (uncorrected p < 0.001), and the significance of connected components was determined via 5,000 permutations against an empirical null distribution of the largest component size, with a corrected significance level of p < 0.05. The permutation procedure randomly shuffled group labels to generate an empirical null distribution under the assumption of exchangeability, with the largest connected component size extracted at each permutation to control for multiple comparisons at the network level. We note that this NBS framework relies on t-statistics derived from a general linear model that assumes homoscedasticity. Should the ADHD and HC groups differ in their conditional variances—plausibly given clinical heterogeneity and the motion-exclusion imbalance—the standard t-based permutation test may not be fully robust to such violations. Robust permutation alternatives using Wald statistics have been proposed to handle heteroscedasticity in neuroimaging GLMs (38, 39). In the absence of formal tests confirming equal variances across groups and covariate strata, the present results should be interpreted cautiously, and future work should consider robust permutation approaches to validate these network-level findings. And finally, BrainNet Viewer v1.7 (http://www.nitrc.org/projects/bnv/) was used for visualization (40).

For brain–behavior associations, two-tailed Pearson partial correlations (controlling for age, sex, and IQ) were performed between FC values and clinical/EF measures within the ADHD group, as HCs showed minimal variance in clinical symptoms. We acknowledge that, although partial correlations and standardized regression coefficients are statistically equivalent in terms of significance testing, the underlying directional question—how brain FC predicts behavior—maps more directly onto an unstandardized regression framework. Future confirmatory analyses may preferentially report unstandardized regression coefficients (B values) to quantify the expected change in behavior per unit increase in FC, controlling for covariates. False Discovery Rate (FDR) correction was applied across all correlation tests; q < 0.05 was considered significant, and 0.05 ≤ q < 0.10 was considered trend-level. All behavioral analyses were conducted in SPSS v26.

## Result

### General demographic information and neuropsychological test data

The differences between the two groups in terms of sex (*p*<0.060), age (*p*<0.541), and intelligence (verbal: *p*<0.543, performance: *p*<0.292, and full-scale: *p*<0.334) were not statistically significant. In terms of CPRS, there were significant statistical differences between the ADHD and HCs in five factor average scores: impulsivity/hyperactivity(*p*<0.001), hyperactivity index (*p*<0.001), psychosomatic disorder (*p*<0.001), conduct problem (*p*<0.001), and learning problem (*p*<0.001). There was no statistically significant difference in anxiety factors (*p*<0.161) between the two groups. In the IVA-CPT, the ADHD showed significantly lower levels of visual control (*p*<0.001), auditory control (*p*<0.006), comprehensive control (*p*<0.001), visual attention (*p*<0.001), auditory attention (*p*<0.001), and comprehensive attention (*p*<0.001) compared to the HCs.

Due to technical limitations, data from the congruent condition of the Stroop task were not available for interference effect computation. Therefore, we report performance on the incongruent condition as an indicator of inhibitory control demand under high-conflict conditions. This limitation should be addressed in future studies by computing the standard Stroop interference effect. There were significant differences (p<0.05) between the ADHD and the HCs in terms of the number of correct responses (*p*<0.004), the number of omissions (*p*<0.014), and reaction time (*p*<0.001). There was no significant difference in the number of errors (*p*<0.822) between the two groups. General demographic information and neuropsychological test data are presented in **Table 2**. These behavioral analyses served two purposes: (1) matching verification—to confirm group comparability, and (2) phenotypic validation—to establish expected clinical deficits.

**Table 2:** Demographic and neuropsychological data and between-group comparisons of ADHD and HCs.

Variable	Groups, mean ± SD		
	ADHD(N = 62)	HCs(N = 46)	*P* value
Age (years)	8.92 ± 2.36	9.24 ± 1.72	0.541
Sex (M/F)	46:16	24:20	0.060^a^
IQ			
Verbal IQ	130.29 ± 13.35	130.46 ± 16.17	0.543
Performance IQ	107.51 ± 12.76	108.70 ± 12.97	0.292
Full-scale IQ	121.45 ± 11.09	123.02 ± 12.54	0.334
CPRS (average score)			
Impulsivity/hyperactivity	1.46 ± 0.67	0.52 ± 0.59	<0.001
Hyperactivity index	1.39 ± 0.53	0.85 ± 0.96	<0.001
Psychosomatic disorder	0.38 ± 0.38	0.17 ± 0.24	0.001
Conduct problem	1.05 ± 0.47	0.65 ± 0.79	0.001
Learning problem	1.75 ± 0.66	0.72 ± 0.69	<0.001
Anxiety	0.42 ± 0.44	0.32 ± 0.25	0.161
IVA-CPT(Quotient)			
Visual control	70.20 ± 28.09	88.87 ± 20.82	<0.001
Auditory control	72.72 ± 25.27	85.37 ± 19.90	0.006
Comprehensive control	69.78 ± 24.20	85.50 ± 20.82	0.001
Visual attention	73.48 ± 24.36	90.00 ± 21.64	<0.001
Auditory attention	74.00 ± 23.41	93.13 ± 20.61	<0.001
Comprehensive attention	71.87 ± 22.67	91.15 ± 22.39	<0.001
Stroop test			
Number of correct responses	43.15 ± 16.78	54.24 ± 18.65	0.004
Number of errors	34.02 ± 10.28	33.52 ± 10.22	0.822
Number of omissions	44.08 ± 21.70	33.15 ± 16.86	0.014
Reaction time (ms)	3100.54 ± 791.51	2328.42 ± 1293.48	0.001

M, male; F, female; ADHD, attention deficit/hyperactivity disorder; HCs, healthy controls; N, number of people; CPRS, Chinese version of the Conner Symptom Scale for Parents; IVA-CPT, Audio Visual Integration Continuous Performance Test. ms--milliseconds. P values were obtained using two-sample t-tests. P^a^ was obtained using the chi-square test.

### Analysis of FC results in the resting state of brain networks

**Figure 1:**
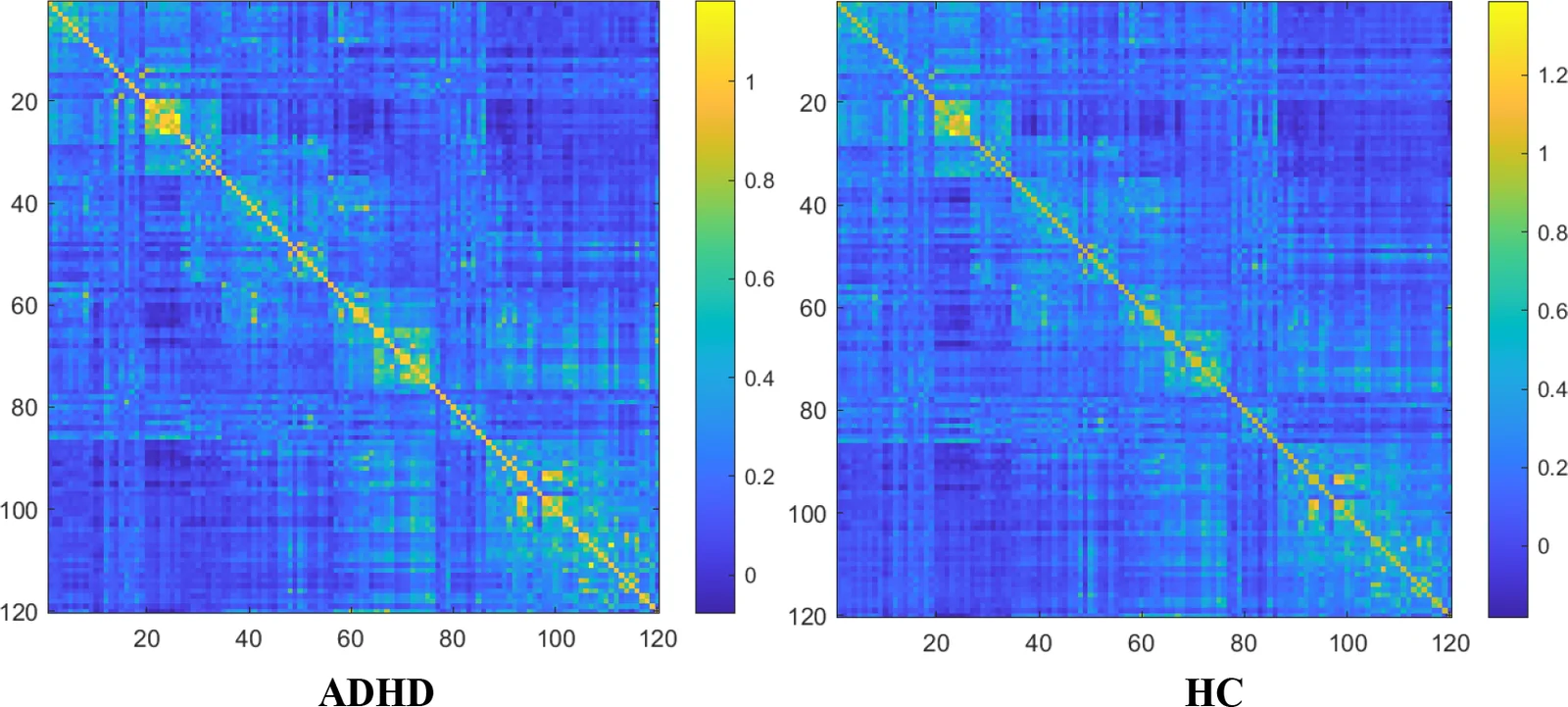
Group-averaged resting-state FC matrices for the ADHD and HC groups. The left heatmap displays the mean Fisher z-transformed ROI-to-ROI correlation matrix for the ADHD group, and the right heatmap displays the corresponding matrix for the HC group. These group-averaged matrices are presented for visualization purposes only; they underlie the group contrast reported in the main text but do not themselves represent statistical test results. Significant between-group differences (NBS-corrected p < 0.05) are detailed in **Table 3** and **Figure 2**.

**Figure 2:**
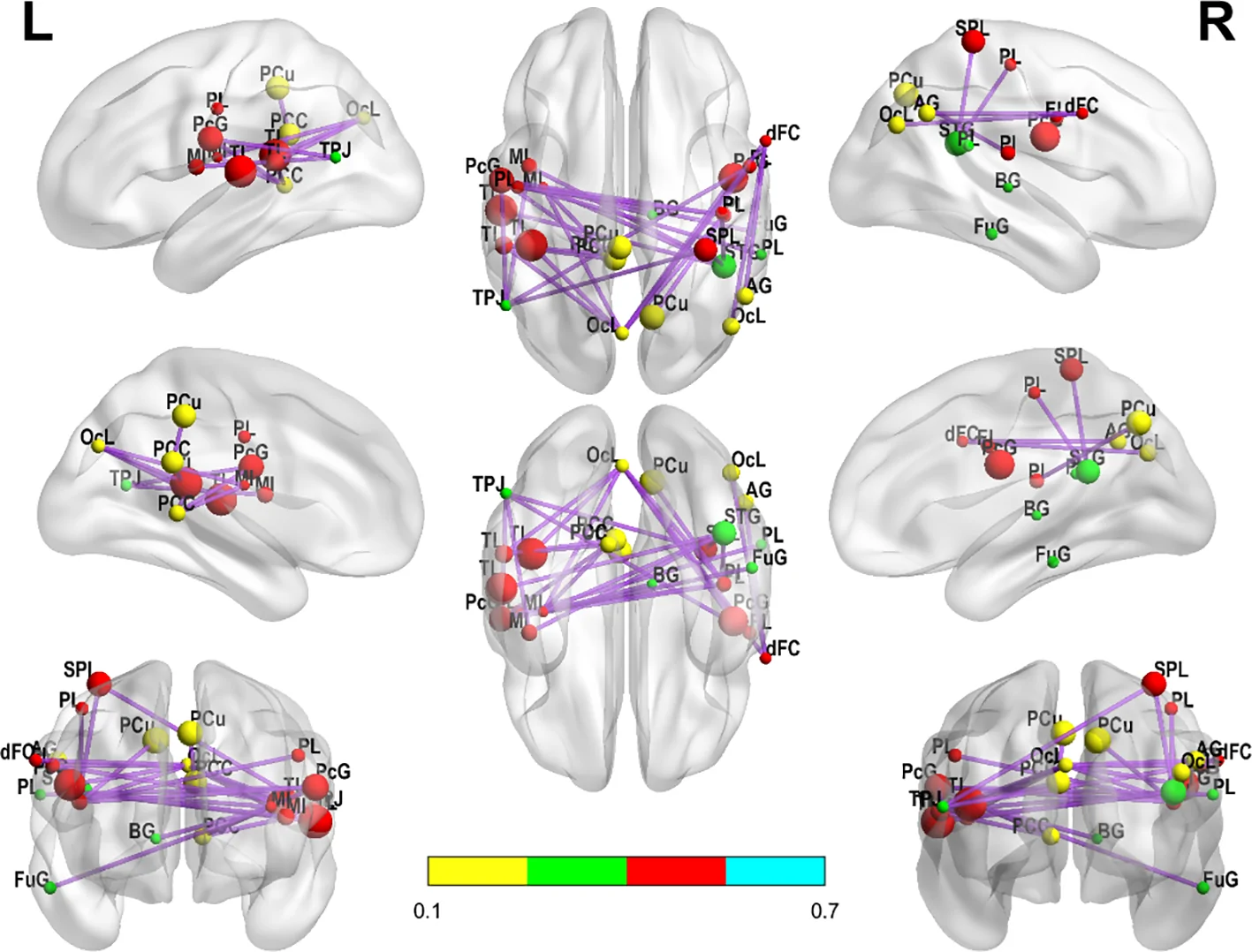
Functional connectivity diagram of resting brain network in ADHD group. Yellow, DMN, Green, SN, Red, SMN, Blue, FPN; L, left; R, right; PCC, posterior cingulate cortex; FuG, fusiform gyrus; PCu, precuneus regions; OcL, occipital lobe; AG, angular gyrus; dFC, dorsal frontal cortex; AI, anterior insula; BG, basal ganglia; MI, middle insula; PI, posterior insula; TL, temporal lobe; PL, Parietal lobe; STG, superior temporal gyrus; TPJ, temporal parietal junction; FL, frontal lobe; PcG, precentral gyrus; SPL, superior parietal lobe.

Compared to HCs, the ADHD group showed significantly enhanced FC within the SMN (right posterior insula to left precentral gyrus and left parietal lobe), between SMN and DMN (involving precentral gyrus, dorsal frontal cortex, temporal lobe, angular gyrus, posterior cingulate cortex, and precuneus), and between SMN and SN (involving middle insula, superior parietal lobule, superior temporal gyrus, basal ganglia, and fusiform gyrus). No statistically significant decreases in FC were found within or between the SMN, DMN, and SN. These statements are not contradictory: the former identifies specific edges where ADHD exhibited hyperconnectivity, whereas the latter indicates that no connected component showed significant hypoconnectivity at the NBS-corrected threshold. No significant statistical differences were found in the FC between SMN and FPN. Further results from the two-sample t-test analysis of resting-state FC are provided in **Table 3**; **Figures 1** and **2**.

**Table 3:** Regions with significantly increased FC in the brain network of ADHD.

Seed region (SMN)	Connected region	Connected network
Right posterior insula	Left precentral gyrus	SMN
	Left parietal lobe	SMN
Left precentral gyrus	Left posterior cingulate cortex	DMN
Right dorsal frontal cortex	Right angular gyrus	DMN
	Left occipital lobe	DMN
	Right occipital lobe	DMN
Right posterior insula	Right occipital lobe	DMN
	Right precuneus	DMN
Left temporal lobe	Left posterior cingulate cortex	DMN
	Left occipital lobe	DMN
Left middle insula	Left posterior cingulate cortex	DMN
	Left occipital lobe	DMN
Right frontal lobe	Left occipital lobe	DMN
Right precentral gyrus	Left occipital lobe	DMN
Left middle insula	Right superior temporal gyrus	SN
	Left temporal parietal junction	SN
	Right basal ganglia	SN
	Right fusiform gyrus	SN
Right frontal lobe	Left temporal parietal junction	SN
Left precentral gyrus	Right parietal lobe	SN
	Left temporal parietal junction	SN
Left temporal lobe	Right superior temporal gyrus	SN
Right parietal lobe	Right superior temporal gyrus	SN
Right superior parietal lobe	Right superior temporal gyrus	SN
	Left temporal parietal junction	SN

SMN, sensorimotor network; DMN, default mode network; SN, salience network. All connections significant at NBS-corrected p < 0.05.

### Correlation analysis between SMN related resting state FC and behavioral as well as cognitive assessment parameters

We conducted partial correlation analysis between FC values and behavioral/cognitive measures within the ADHD group, controlling for age, sex, and full-scale IQ, and performed multiple comparison correction. **Table 4** summarizes the results. We acknowledge several statistical limitations of our approach. First, the use of univariate partial correlations, even with FDR correction, ignores the shared variance among FC edges and behavioral measures and cannot parse the relative contribution of multiple correlated predictors. Future studies should employ multivariate predictive frameworks (e.g., partial least squares, ridge, or LASSO regression with nested cross-validation) to validate these exploratory findings (41, 42). Second, FDR correction is less stringent than familywise error rate (FWER) control; had FWER been applied (e.g., Bonferroni or Holm–Bonferroni), fewer associations would likely survive correction. We report FDR-corrected results as exploratory and emphasize that all brain–behavior correlations require independent replication with stricter error control or pre-registered hypotheses. Third, the modest sample size (N = 108 after exclusion) limits statistical power and generalizability; validation in larger, population-based cohorts (e.g., ABCD, UK Biobank) is essential (41, 42).

**Table 4:** Partial correlation analysis results of FC in the brain networks in ADHD.

FC pair	Clinical/EF measure	r	p	*q* (FDR)
Intra-SMN				
Right posterior insula - Left parietal lobe	Impulsivity/hyperactivity	-0.35	0.006	0.028
	Hyperactivity index	-0.33	0.011	0.050†
SMN-DMN				
Left precentral gyrus - Left posterior cingulate cortex	Impulsivity/hyperactivity	-0.37	0.004	0.018
	Hyperactivity index	-0.34	0.01	0.045
Right dorsal frontal cortex - Right occipital lobe	Visual Control	0.41	0.001	0.009
	Auditory control	0.34	0.008	0.036
	Comprehensive control	0.41	0.001	0.009
SMN-SN				
Left temporal lobe – Right superior temporal gyrus	Correct responses	-0.34	0.009	0.041
	Omission errors	0.31	0.017	0.051†

SMN, sensorimotor network; DMN, default mode network; SN, salience network. Bold q-values indicate FDR-corrected significance (q < 0.05). † Trend-level significance (0.05 ≤ q < 0.10).

**Figure 3:**
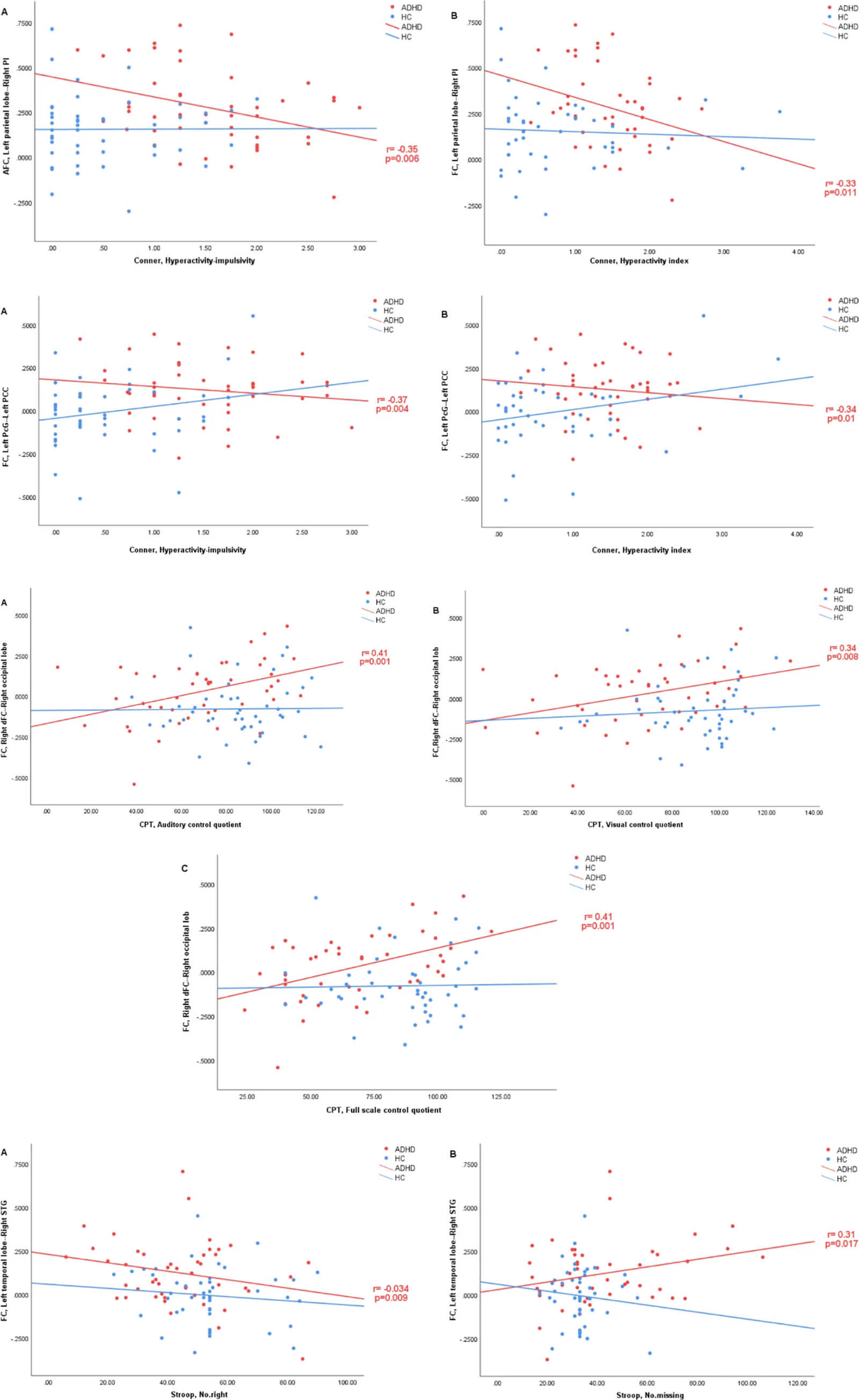
**3–1** Correlation between the FC between the left parietal lobe and the right PI and CPRS. Figures A and B represent the partial correlation analysis results between FC within the SMN (between the left parietal lobe and the right PI) and the CPRS hyperactivity/impulsivity average score and hyperactivity index in children with ADHD, respectively. **3–2** Correlation between the FC between the left PcG and the left PCC and CPRS. Figures A and B represent the partial correlation analysis results between FC between SMN and DMN (left PcG and left PCC) and the CPRS hyperactivity/impulsivity average score and hyperactivity index in children with ADHD, respectively. **3–3** Correlation between the FC between the right dFC and the right OcL and IVA-CPT. Figures A, B, and C represent the partial correlation analysis results of FC between SMN and DMN (right dFC and occipital lobe) and the IVA-CPT auditory control, visual control, and comprehensive control quotient in children with ADHD, respectively. **3–4** Correlation between the FC between the left temporal lobe and the right STG and Stroop. Figures A and B represent the partial correlation analysis results between the FC between SMN and SN (left temporal lobe and right STG) and Correct responses and Omission errors in Stroop test in children with ADHD, respectively. .

Intra-SMN Connectivity: Enhanced FC between the right posterior insula and left arietal lobe was negatively associated with CPRS impulsivity/hyperactivity (*q*<0.028) and showed a trend-level negative association with hyperactivity index (*q*<0.050) (**Figure 3–1**).
SMN–DMN Connectivity: First, enhanced FC between the left precentral gyrus and left posterior cingulate cortex was negatively associated with CPRS impulsivity/hyperactivity (*q*<0.018) and hyperactivity index (*q*<0.045) (**Figure 3–2**). Second, enhanced FC between the right dorsal frontal cortex and right occipital lobe showed positive associations with IVA-CPT control quotients: visual (*q*<0.009), auditory (*q*<0.036), and comprehensive control (*q*<0.009) (**Figure 3–3**).
3. SMN–SN Connectivity: Enhanced FC between the left temporal lobe and right superior temporal gyrus was negatively associated with Stroop correct responses (*q*<0.041) and positively associated with omissions (*q*<0.051, trend-level) (**Figure 3–4**).

## Discussion

The present study investigated resting-state FC patterns of the SMN in medication-naïve children with ADHD. We report three main findings. First, children with ADHD showed significantly increased FC within the SMN (right posterior insula to left precentral gyrus/parietal lobe) compared to HCs. Second, ADHD was characterized by enhanced FC between the SMN and the DMN (precentral gyrus, dorsal frontal cortex, temporal lobe, angular gyrus, posterior cingulate cortex, precuneus) and between the SMN and the SN (insula, superior parietal lobule, superior temporal gyrus, basal ganglia, fusiform gyrus). Third, within the ADHD group, these connectivity alterations showed distinct directional associations with clinical and cognitive profiles: increased intra-SMN and SMN–DMN FC were associated with reduced hyperactivity/impulsivity and better response control, whereas elevated SMN–SN FC was associated with poorer inhibitory control on the Stroop task. We emphasize that these associations are framed in predictive terms (i.e., FC as a predictor of behavior) to align with the directional nature of the research question, while noting that the cross-sectional design precludes causal inferences.

### Intra-SMN functional connectivity in ADHD

Our observation of increased intra-SMN FC in ADHD aligns with prior reports of altered sensorimotor functional activity in drug-naïve pediatric samples (10, 11). However, the clinical correlates require cautious interpretation. We found that stronger FC between the right posterior insula and the left parietal lobe was cross-sectionally associated with lower CPRS impulsivity/hyperactivity scores. This association does not establish causality; it may reflect (a) individual differences in baseline neural organization among children with milder symptoms, (b) developmental variation across the 6–15 year age span, or (c) a distinct neurobiological subtype characterized by both enhanced intra-SMN coupling and milder behavioral expression. Longitudinal and intervention designs are needed to determine whether this pattern actively mitigates symptoms or merely co-occurs with them.

These findings appear discrepant with Chen et al. (27), who reported that enhanced inter-hemispheric sensorimotor FC was associated with greater motor overflow in children with ADHD. This divergence likely stems from methodological differences: Chen et al. employed ICA to examine inter-hemispheric somatomotor subnetwork (SSN) connectivity, whereas our seed-based approach focused on intra-SMN modulation involving the posterior insula and parietal lobe. It is plausible that inter-hemispheric disinhibition and intra-network modulation represent distinct neural mechanisms with divergent behavioral consequences. Similarly, Sörös et al. (25) found increased adult SMN FC linked to greater hyperactivity severity; however, direct comparison is constrained by developmental stage, sex distribution, and medication status. In contrast, McLeod et al. (20) reported reduced FC between M1 and somatosensory/striatal regions in children with ADHD, half of whom were medicated. Taken together, the SMN connectivity literature in ADHD is marked by heterogeneity in directionality, analytic approach, and clinical correlates, underscoring the need for converging methodologies in well-characterized, medication-naïve pediatric cohorts.

### SMN–DMN and SMN–SN connectivity

Enhanced SMN–DMN FC was negatively correlated with parent-rated hyperactivity/impulsivity and positively correlated with IVA-CPT control quotients. This pattern suggests that more efficient intrinsic coordination between sensorimotor and default-mode systems may co-occur with milder symptoms and better cognitive performance, although the cross-sectional design precludes inferences regarding directionality. These results converge with Luo et al. (18), who reported that increased dynamic FC between DMN and SMN was associated with reduced ADHD symptoms and better behavioral inhibition in children. However, Vatansever et al. (24) observed reduced SMN–DMN FC in relation to subclinical ADHD symptoms in healthy young adults. Such discrepancies may reflect developmental differences: adult network maturation and medication history may reshape DMN–SMN interactions in ways that differ from medication-naïve children. Future studies employing both resting-state and task-state designs in the same participants are needed to clarify whether enhanced resting-state coupling facilitates more efficient task-state network switching or whether it represents a stable trait marker.

It is also noteworthy that recent large-scale mega-analytic studies have implicated dorsal attention–DMN connectivity in ADHD diagnosis and attention problems. Norman et al. (43) analyzed over 2,600 participants across multiple cohorts and found that ADHD diagnosis was associated with less anticorrelation between the default mode and task-positive networks (including somatomotor, salience/ventral attention, and dorsal attention networks), with the largest effect size observed for DMN–dorsal attention connectivity (partial-*r* = 0.06). Similarly, Duffy and Helwig (42), using ABCD baseline data (N≈9,000), demonstrated that increased integration between default mode and task-relevant networks predicted attention problems in children. These convergent findings suggest that interactions between sensory/attention networks and the DMN may represent a general mechanism underlying ADHD pathophysiology that warrants integration with SMN-focused models.

Conversely, elevated SMN–SN FC was associated with poorer Stroop performance (fewer correct responses, more omission errors). The SN is critical for detecting salient internal and external stimuli and regulating sensory-motor responses (44). Stronger SMN–SN coupling may reflect heightened sensitivity to task-irrelevant stimuli that interferes with sensorimotor integration and response control. This interpretation is consistent with Lin et al. (23) and Schwarzlose et al. (28), who reported increased SMN–SN FC in medication-naïve adults with ADHD and in children with sensory over-responsivity, respectively. Nevertheless, because these associations are modest and cross-sectional, they should be viewed as exploratory. Causal interpretations—such as whether heightened salience detection drives executive dysfunction or whether both arise from a common underlying factor—are not supported by the current data and require replication with stricter error control or pre-registered hypotheses.

### Methodological considerations and limitations

Several limitations should be considered when interpreting these findings. First, the cross-sectional design precludes causal inferences; longitudinal studies are required to determine whether the observed FC patterns represent stable traits, developmental adaptations, or state-dependent fluctuations. Second, we excluded 11 ADHD participants due to excessive head motion versus only 3 HCs. Because hyperactivity increases in-scanner movement, this imbalance may introduce survivorship bias. We chose exclusion over advanced motion-correction strategies (e.g., ICA-AROMA, multi-echo acquisition) to maintain conservative data quality standards, but future studies should employ these techniques to retain more representative data. Third, our strict inclusion criteria (IQ > 90, no medication history, right-handedness) yielded a relatively high-functioning sample. Given the existence of publicly available large samples (e.g., ABCD, UK Biobank), our modest sample size (N = 108 after exclusion) should be viewed as a pilot-scale investigation requiring validation in population-based cohorts. Fourth, the children spanned a wide age range (6–15 years) encompassing considerable neurodevelopmental change. While age was included as a covariate, this approach cannot fully capture non-linear developmental trajectories, age-by-diagnosis interactions, or the potentially different maturational trajectories of SMN connectivity across childhood and adolescence. FC patterns and their clinical correlates may differ across this age range, and our cross-sectional design cannot disentangle developmental effects from individual differences. This limitation hinders generalizability due to having a relatively small number at each age and not properly incorporating age-related differences into the statistical modeling. Future studies should examine age-stratified subgroups or employ longitudinal designs to clarify developmental heterogeneity in SMN connectivity. Fifth, NBS controls multiple comparisons at the network level but does not fully capture the significance of individual nodes; complementary node-wise or multivariate predictive frameworks (e.g., partial least squares, ridge regression with nested cross-validation) would better leverage the correlated structure of FC data and improve generalizability, particularly given our modest sample size (N = 108 after exclusion). Sixth, the brain–behavior correlations were corrected using FDR rather than familywise error rate; these findings should therefore be interpreted as exploratory and require independent replication. Seventh, the NBS permutation procedure employed t-statistics under a standard GLM that assumes homoscedasticity. If the ADHD and healthy control groups differed in their conditional variances—plausibly given the clinical heterogeneity of ADHD and the between-group imbalance in motion-related exclusions—this assumption may be violated. Robust nonparametric tests using Wald statistics offer a more general alternative when variance structures differ across groups or covariate levels. We therefore acknowledge this as a potential methodological limitation and recommend that independent replication employ robust permutation frameworks to confirm the present network-level inferences. Eighth, we have not yet provided open data and analysis code. To ensure reproducibility, we will make de-identified FC matrices and analysis scripts available upon reasonable request, subject to ethical approvals governing pediatric clinical data.

## Conclusion

In this study, we used rs-fMRI to compare SMN-related FC between medication-naïve children with ADHD and HCs. Our findings indicate that ADHD is characterized by increased FC within the SMN and between the SMN and the DMN and SN. These altered connectivity patterns showed distinct associations with clinical and executive function measures. Specifically, (1) increased intra-SMN FC was cross-sectionally associated with reduced hyperactivity/impulsivity severity, though whether this reflects developmental variation, individual differences, or a neurobiological subtype remains to be determined; (2) enhanced SMN-DMN FC was associated with milder hyperactive/impulsive symptoms and better executive performance, consistent with more efficient intrinsic network coordination, though directionality cannot be inferred from these cross-sectional data; and (3) elevated SMN-SN FC was associated with greater executive dysfunction, potentially indicating that heightened salience detection interferes with sensorimotor integration and response control. We hope these findings contribute to refining theoretical models of ADHD brain networks and provide new clues for clinical diagnosis, intervention targeting, and future mechanistic research.

## Data Availability

The raw data supporting the conclusions of this article are not publicly available due to ethical and privacy restrictions related to pediatric clinical data. De-identified data and analysis scripts will be made available by the corresponding author (Chuang Yang, dryangchuang@163.com) upon reasonable request, subject to approval by the Ethics Committee of the First Affiliated Hospital of Wenzhou Medical University.
